# A Novel Surgical Approach for Double-Outlet Right Ventricle With Noncommitted Ventricular Septal Defect

**DOI:** 10.1016/j.atssr.2025.12.005

**Published:** 2025-12-25

**Authors:** Mélanie Frei, Tomasz Nalecz, Alexandre Pelouze, Jalal Jolou, Maurice Beghetti, Julie Wacker, Tornike Sologashvili

**Affiliations:** 1 Cardiovascular Surgery Department, Geneva University Hospital, Geneva, Switzerland; 2 Pediatric Cardiology Unit, Department of Women, Child and Adolescent Medicine, Geneva University Hospitals and Faculty of Medicine, Geneva, Switzerland

## Abstract

Double-outlet right ventricle (DORV) is a highly variable heart disease. Biventricular repair of noncommitted ventricular septal defect (VSD) forms of DORV is often overlooked in favor of palliative surgery such as Fontan. We present the case of a 7-year-old patient, with the diagnosis of noncommitted VSD DORV, who underwent biventricular correction, with good short-term result. The management of complex DORV with noncommitted VSD is evolving. Previously oriented toward Fontan palliation, it now benefits from surgical advances allowing biventricular repair, reflecting surgical advances preserving optimal heart physiology.

Double-outlet right ventricle (DORV) is a rare and complex congenital heart disease, representing ∼1% to 3% of all congenital heart defects, in which the aorta and pulmonary artery emerge predominantly or entirely from the right ventricle (RV).^1^ DORV is classified into 4 major subtypes based on the location of the ventricular septal defect (VSD) relative to the great vessels: subaortic, subpulmonary, double committed, and noncommitted.^2^

The complexity of surgical treatment for DORV arises from its significant anatomical variability, including the spatial relationship of the great vessels and the size and location of the VSD relative to them. Among the subtypes, DORV with a noncommitted VSD is considered one of the most challenging, due to the remoteness of the VSD, which is not aligned with either great vessel, making the creation of a left ventricular-aortic baffle anatomically difficult, with increased risk of subaortic obstruction, valvular distortion, and long-term ventricular dysfunction.^3^, ^4^, ^5^

In such complex cases, Fontan palliation is often performed. Although effective in ensuring survival, this strategy imposes a nonphysiological circulation that predisposes patients to chronic complications such as liver damage, lymphatic disorders, arrhythmias, and progressive ventricular dysfunction.^6^

We report a complex case of DORV with a noncommitted VSD in which successful biventricular repair was achieved, highlighting the feasibility of anatomical correction in selected patients and underscoring the importance of individualized surgical planning.

A 7-year-old female patient was referred with a diagnosis of DORV and a noncommitted VSD. Her oxygen saturation was ∼85% on room air. Echocardiography demonstrated DORV with side-by-side great arteries, with a slightly anterior-right aorta and a posterior-left pulmonary trunk. The RV was severely hypertrophied and moderately dilated, whereas the left ventricle (LV) appeared small and hypertrophied, and preserved biventricular systolic function. A medium-size atrial septum defect was present.

A noncommitted VSD was identified, precluding the possibility of tunneling to the great vessels without risk of obstruction. The atrioventricular and semilunar valves were competent, although the mitral and pulmonary annuli were of small caliber. Severe subvalvular pulmonary stenosis was noted, secondary to posterior deviation of the conal septum, with gradient >100 mm Hg. The pulmonary trunk and its branches were of normal size. A computed tomographic scan confirmed the echocardiographic findings (Figure 1).

**Figure 1:**
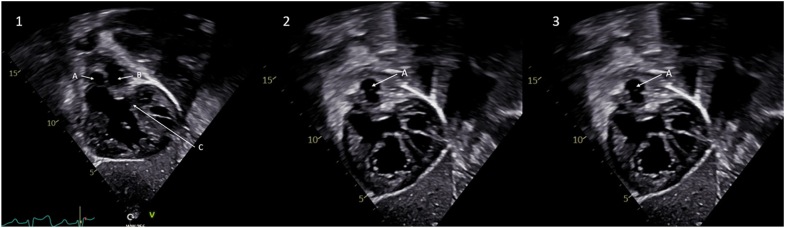
Preoperative echocardiography shows the (1) aorta (A), pulmonary artery (B), and ventricular septal defect (VSD) (C); (2) the inlet VSD (A) and (3) the pulmonary artery (A) far away from the VSD.

The patient underwent complete repair with LV tunneling into the aortic valve. A right atriotomy was performed to evaluate anatomy, confirming the presence of a noncommitted VSD. An aortotomy was performed to expose the aortic valve.

Given the complexity of the anatomical configuration, we used a novel approach, using a partial aortic root detachment, to optimize exposure of intracardiac structures and the VSD, thereby facilitating LV-to-aorta tunneling and enabling effective management of straddling morphologies.

The procedure began with dissection and harvesting of the right coronary button from the right coronary sinus, which in this case also gave rise to the left anterior descending artery. Mobilization was extended 2 to 3 cm along the coronary artery to create enough space at the level of the aortic root (Figure 2.1).

**Figure 2:**
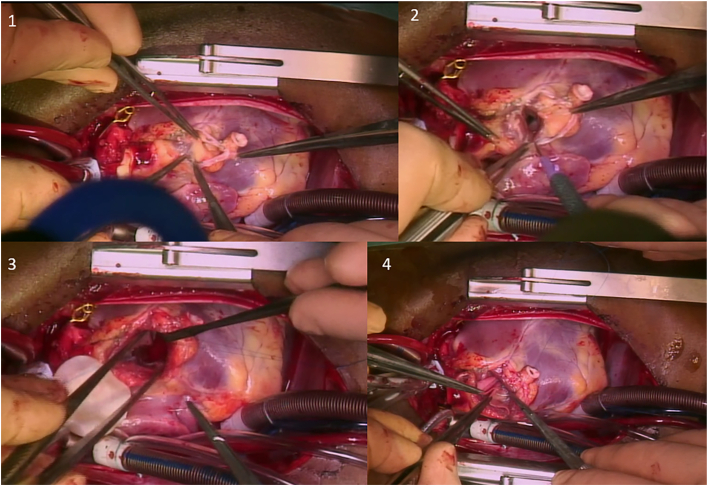
Intraoperative view shows (1) harvest of the right coronary button, (2) detachment of the aortic root, (3) the ventricular septal defect, and (4) tunneling of left ventricle to the aortic valve with 2 patches.

A circumferential incision parallel to the aortic anulus was performed, leaving approximately a 5-mm muscular rim for subsequent resuturing. The aortic root was detached at nearly 50% of the annulus, providing direct access to the VSD (Figure 2.2). Through this incision, hypertrophied muscle bundles within the RV outflow tract were resected, relieving the obstruction. The VSD was then enlarged toward the aortic valve, accompanied by resection of septal muscle tissue to optimize the outflow pathway (Figure 2.3).

Tunneling the LV to the aortic valve was achieved using 2 patches to prevent tortuosity (Figure 2.4). The ventriculotomy was closed directly at the level of the aortic annulus, with attachment of the annulus to the RV using the sandwich technique and a biological patch to complete closure of the VSD. The right coronary button was reimplanted into its original position at the aortic root. Through the right atriotomy, the atrial septum defect was closed directly. A longitudinal incision of the pulmonary trunk was then performed, followed by commissurotomy and resection of fibrotic tissue through the pulmonary valve. Subsequently, a right infundibulectomy was performed, with resection of hypertrophied muscle bundles and closure of the infundibulum. The aortic cross-clamp was removed, and cardiopulmonary bypass was weaned.

At 1 month, echocardiography demonstrated satisfactory results, with preserved biventricular systolic function, unobstructed LV outflow tract, moderate pulmonary insufficiency, and mild mitral insufficiency (Figure 3).

**Figure 3:**
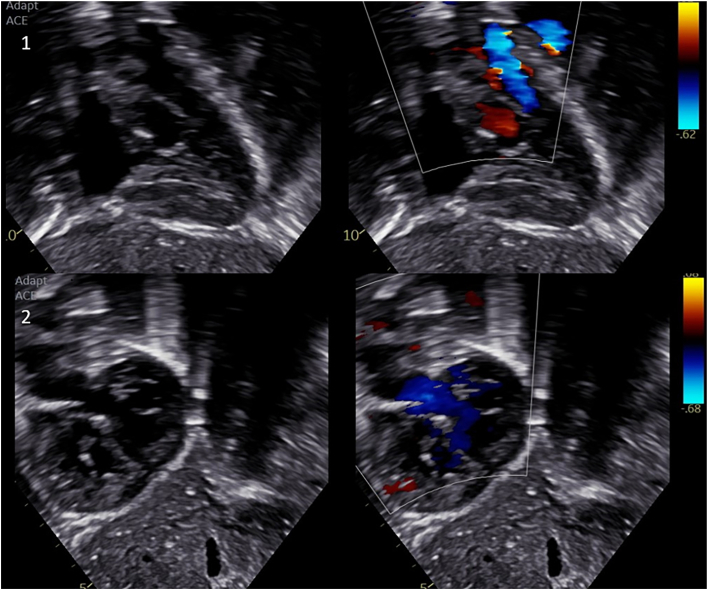
Postoperative echocardiography shows the (1) right ventricular outflow tract and (2) left ventricular outflow tract.

## Comment

Management of DORV with noncommitted VSD represents a major challenge in congenital cardiac surgery. Traditionally, this complex malformation was treated primarily with a palliative strategy. However, advances in surgical techniques and a better understanding of cardiac anatomy are now challenging this conventional approach.

This surgical case aligns with findings reported in the literature. Several groups have demonstrated the feasibility of biventricular repair, even in anatomies initially considered “unrepairable.” Barbero-Marcial and colleagues^3^ pioneered the use of multiple intraventricular patches to redirect LV flow toward the aorta, achieving favorable postoperative survival with a low risk of tunnel obstruction. Lacour-Gayet and colleagues^4^ refined this technique by combining intraventricular rerouting with arterial switch in selected cases, thereby improving flow direction and reducing anatomical constraints. More recently, Li and colleagues^5^ confirmed the feasibility of biventricular repair in experienced centers, with satisfactory outcomes in survival.

Nevertheless, biventricular correction remains technically demanding, and not all patients are eligible for this approach. Fontan palliation continues to be a proven alternative, particularly when the LV is hypoplastic or when rerouting is not feasible.^6^ However, the significant late complications associated with univentricular palliation justify the ongoing pursuit of biventricular solutions whenever possible.

Our case illustrates a novel surgical technique that provides improved access to intracardiac structures, especially in complex anatomies or in the presence of associated anomalies such as straddling. This approach enables the creation of a well-aligned tunnel under direct vision, minimizing the risk of obstruction and residual VSDs as well as preserving 2 native valves avoiding more surgery in the future, unlike other biventricular techniques. Furthermore, it appears less traumatic to the myocardium, because fewer muscle resections are required. The favorable outcome observed at 1 month postoperatively is consistent with the current trend in pediatric cardiac surgery, which emphasizes maximizing biventricular physiology even in complex anatomical settings, through innovative surgical strategies and meticulous preoperative planning.

In conclusion, evolution of surgical techniques enables biventricular repair in DORV with noncommitted VSD, a lesion traditionally managed with Fontan palliation. This favorable early outcome reflects an important shift in congenital cardiac surgery toward prioritizing biventricular physiology whenever possible.

## Declaration of Generative AI And AI-Assisted Technologies in The Writing Process

During the preparation of this work the authors used ChatGPT to improve readability. After using this tool, the authors reviewed and edited the content as needed and take full responsibility for the content of the publication.
